# Complication of a Swan–Ganz catheter: an intravascular knot

**DOI:** 10.1093/ehjcr/ytad543

**Published:** 2023-11-06

**Authors:** Ronan Canitrot, Thibault Lhermusier, Clément Servoz

**Affiliations:** Department of Cardiology, Rangueil University Hospital, 1 Avenue Jean Poulhés, TSA 50032, Toulouse, Cedex 9 31059, France; Department of Cardiology, Rangueil University Hospital, 1 Avenue Jean Poulhés, TSA 50032, Toulouse, Cedex 9 31059, France; Department of Cardiology, Rangueil University Hospital, 1 Avenue Jean Poulhés, TSA 50032, Toulouse, Cedex 9 31059, France

We describe the case of a 59-year-old woman admitted for an arterial pulmonary hypertension exploration. A transthoracic echocardiography revealed a preserved left ventricular ejection fraction and an arterial pulmonary hypertension with a systolic pulmonary arterial pressure estimated at 70 mmHg.

To confirm the diagnosis, a right heart haemodynamic catheterization was performed with a Swan–Ganz catheter. Under ultrasound guidance, the right internal jugular vein was punctured. Upon completing the diagnostic procedure and obtaining the haemodynamic parameters, removing the Swan–Ganz catheter appeared impossible.

A chest X-ray revealed an endovascular loop and a knot formed in the superior vena cava, rendering its removal impossible. After knot visualization, interventional catheterization technique was selected to extract the Swan–Ganz catheter (*Figure 1A*).

**Figure 1:**
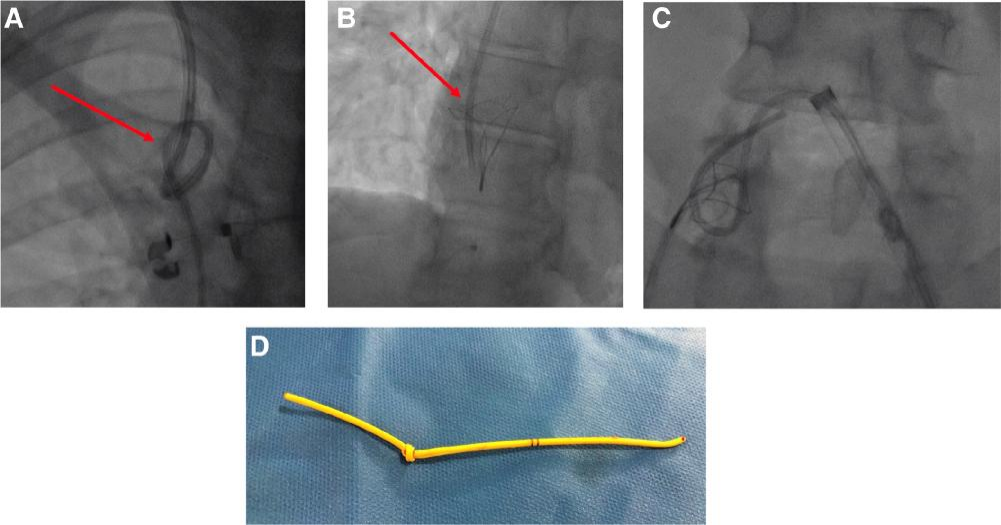
Interventional catheterization technique for the retrieval of the Swan–Ganz catheter. (*A*) Endovascular knot formed in the superior vena cava. (*B*) Using the lasso technique to recover the Swan–Ganz catheter. (*C*) Swan–Ganz catheter was retrieved through the left femoral vein. (*D*) Success of the procedure.

First, the Swan–Ganz catheter was cut at the proximal segment of the internal jugular vein, enabling its subsequent extraction through a femoral access. Then, a 6F introducer sheath was placed on the right femoral vein. The first operator manoeuvred and secured the cut Swan–Ganz catheter via the jugular access, while a second operator attempted to capture it through the femoral access. The Swan–Ganz catheter knot was successfully captured using the lasso technique (EN Snare 6F) (*Figure 1B*; see Supplementary material online, *Video S1*). Under ultrasound guidance, a 14F introducer sheath, after pre-closing technique, was then placed in the left femoral vein. Through the 14F introducer, the Swan–Ganz catheter was retrieved (*Figure 1C* and *D*; see Supplementary material online, *Video S2*). A double approach was used to ensure the possibility of catching and withdrawing the catheter. A second snare (EN Snare 7F) was used to catch and withdraw the catheter through the left femoral vein. A singular vascular approach could be possible for this procedure.

Right heart haemodynamic catheterization complications are rare but potentially serious (vascular complications, thrombosis, arrhythmias, infections, pulmonary artery ruptures).^1^

The formation of an intracardiac or endovascular knot is a rare but severe complication.^2^ In order to prevent this complication, Swan–Ganz exploration should be performed under fluoroscopic guidance. Resistance or difficulties encountered while advancing the Swan–Ganz catheter indicate a potential knot formation. Finally, physicians should acquaint themselves with various strategies to manage knot formation like the use of a guide to untie the knot or balloon inflation in the loop.

We describe a successful snaring technique to retrieve a tightly knotted Swan–Ganz catheter.

## Supplementary Material

ytad543_Supplementary_Data

## Data Availability

The data underlying this article are available in the article and in its online Supplementary material.

## Supplementary material

**Consent:** Consent for publication has been obtained, in line with the COPE best practice guidelines, and that the individual(s) who is being reported on is aware of the possible consequences of that reporting.

**Funding:** None declared.
